# Editorial: Financial anxiety in cancer prevention and control

**DOI:** 10.3389/fpsyg.2023.1304079

**Published:** 2023-10-16

**Authors:** Helen M. Parsons, Matthew P. Banegas, Gil Bar-Sela, Salene M. Jones

**Affiliations:** ^1^Division of Health Policy and Management, School of Public Health, University of Minnesota, Minneapolis, MN, United States; ^2^Department of Radiation Medicine and Applied Sciences, University of California, San Diego, San Diego, CA, United States; ^3^Cancer Center, Emek Medical Center, Afula, Israel; ^4^Public Health Sciences Division, Fred Hutchinson Cancer Center, Seattle, WA, United States

**Keywords:** financial hardship, financial toxicity, cancer, methods, protocol

Financial hardship is commonly experienced by patients after a cancer diagnosis and can impede participation in cancer control activities and reduce access to cancer care and survivorship support (Altice et al., 2017; Yabroff et al., 2020). Cancer-related financial hardship can be substantial, with recent research highlighting that this burden continues to grow as insurers increasingly shift the costs of cancer care to patients and their families (Mariotto et al., 2011; Shih et al., 2017; Laviana et al., 2020). Financial hardship is a complex issue that is experienced uniquely across patients and which can be categorized into three broad domains: material, behavioral and psychological. The psychological aspect of financial hardship is a distinct concept that provides additional context on the economic impact of cancer. Sometimes referred to as financial anxiety, this psychological aspect of financial hardship often includes feelings of worry about an individual's money situation such as their income, job security and an ability to afford healthcare and living expenses (e.g., rent, transportation, food) (Prawitz et al., 2006; Peterson and Miller, 2019). While financial anxiety in cancer care can be particularly troubling and impact financial hardship, it has received less attention than more material and behavioral aspects of financial outcomes after cancer. To help move the field forward and address the role of financial anxiety in cancer, this Research Topic on *Financial anxiety in cancer prevention and control* aims to bring together the latest articles from researchers working in the area of psycho-oncology and financial hardship, focused on financial anxiety, financial worry, and financial stress.

The first set of articles in this Research Topic further examine underlying conceptual models and measure development for understanding financial anxiety. Five studies highlight the need to better understand the concepts comprising financial anxiety as well as opportunities for measuring these concepts after cancer diagnosis. Biddell et al. present a novel conceptual model that includes the protective, modifying and hindering multi-level factors that affect financial anxiety and other dimensions of cancer-related financial hardship. Another paper reports on the work of the Emotional wellbeing and Economic Burden (EMOT-ECON) Research Network. The EMOT-ECON conceptual framework is based on the stress appraisal coping models, emphasizing how people with cancer actively respond to the triggers of financial hardship, using coping strategies that can increase or decrease anxiety. Three additional papers report on the development of financial hardship measures for specific populations: older adults in China; Spanish speakers in the United States; and people with prostate cancer in the United States. These measures show that the factors that contribute to financial anxiety and financial hardship can differ by location, age, and cancer type. While we know much about financial hardship and financial anxiety from cancer, the complexity of this public health problem requires rigorously developed conceptual models and measures such as those reported here.

In addition to new methods for understanding and measuring financial anxiety, this issue also highlights new research into the impact of financial anxiety on cancer care and outcomes. One study by Shi et al. examined the association between financial anxiety of individuals with cancer and acute care visits, finding that those with severely depleted material, practical and social coping resources related to the financial impact of their cancer were at greater risk for repeat acute care visits than individuals with more robust coping resources. A second study examined the role of frailty in medical financial hardship, finding that among older cancer survivors in the US, frail cancer survivors were vulnerable to not only material financial hardship but poorer psychological and behavioral hardship outcomes. These studies add to the growing evidence of the role financial anxiety may play in risk for poor outcomes after cancer diagnosis.

Lastly, for nearly a decade, there has been a wealth of research dedicated to documenting and detailing the vast adverse effects that patients experience from the financial hardships associated with cancer and its treatment–commonly referred to as financial toxicity within the field of oncology. More recently, there have been calls by advocates, experts, and professional organizations to move beyond describing cancer-related financial toxicity, toward testing and identifying effective solutions to intervene on this critical issue. While there is no high-level evidence (e.g., Level I-III) showing the effectiveness of any intervention to address financial toxicity, there are approaches that have emerged as potential solutions and which served as the basis for recent clinical trials, including financial navigation (clinicaltrials.gov ID: NCT05018000; NCT04960787; NCT04931251), health insurance navigation (NCT05829070; NCT04448678; NCT04520061); and workforce communication and job retention (NCT03572374) (Smith et al., 2022). Doherty et al. expand this body of clinical trials targeting cancer-related financial hardship, presenting their protocol for Guaranteed Income and Financial Treatment (GIFT), a large randomized controlled trial to test the effectiveness of unconditional cash transfers (UCTs) on financial toxicity, health-related quality of life, treatment adherence, and other cancer outcomes, among cancer patients who have low incomes. This societal-level intervention targets both federal and state policy and public benefit programs to provide UCTs, adding to the prior individual- and health clinic/system-level interventions being assessed for effectiveness against financial toxicity.

There has been tremendous progress in our understanding of cancer-related financial hardship. While most research has focused on material financial hardship, including bankruptcy and going into debt, psychological aspects such as financial anxiety have received less attention. This Research Topic of Frontiers in Psychology, *Financial Anxiety in Cancer Prevention and Cancer Control*, helps fill this knowledge gap by presenting findings from an impressive variety of studies that underscore the complexity of financial anxiety as a component of financial hardship and which provide frameworks, methods, and interventions to guide future research. Importantly, this Research Topic highlights the need for studies to further elucidate the bidirectional associations between the multiple components of financial hardship with both health and health care outcomes, as well as to develop and test multi-level interventions that either directly or indirectly aim to mitigate the financial hardship from cancer.

## Author contributions

HP: Conceptualization, Writing–original draft. MB: Conceptualization, Writing–original draft. GB-S: Writing–review and editing. SJ: Conceptualization, Writing-original draft.
